# Safety, pharmacokinetics, metabolism and radiation dosimetry of ^18^F-tetrafluoroborate (^18^F-TFB) in healthy human subjects

**DOI:** 10.1186/s13550-017-0337-5

**Published:** 2017-10-27

**Authors:** Huailei Jiang, Nicholas R. Schmit, Alex R. Koenen, Aditya Bansal, Mukesh K. Pandey, Robert B. Glynn, Bradley J. Kemp, Kera L. Delaney, Angela Dispenzieri, Jamie N. Bakkum-Gamez, Kah-Whye Peng, Stephen J. Russell, Tina M. Gunderson, Val J. Lowe, Timothy R. DeGrado

**Affiliations:** 1 Department of Radiology, Mayo Clinic, 200 First St. SW, Rochester, MN 55905 USA; 2 Department of Medicine, Mayo Clinic, Rochester, MN USA; 3 Department of Obstetrics and Gynecology, Mayo Clinic, Rochester, MN USA; 4 Department of Molecular Medicine, Mayo Clinic, Rochester, MN USA; 5 Department of Clinical Statistics, Mayo Clinic, Rochester, MN USA

**Keywords:** Sodium/iodide symporter, Tetrafluoroborate, ^18^F-fluorine, PET imaging, Biodistribution, Dosimetry

## Abstract

**Background:**

^18^F-Tetrafluoroborate (^18^F-TFB) is a promising iodide analog for PET imaging of thyroid cancer and sodium/iodide symporter (NIS) reporter activity in viral therapy applications. The aim of this study was to evaluate the safety, pharmacokinetics, biodistribution, and radiation dosimetry of high-specific activity ^18^F-TFB in healthy human subjects.

**Methods:**

^18^F-TFB was synthesized with specific activity of 3.2 ± 1.3 GBq/μmol (at the end of synthesis). Dynamic and whole-body static PET/CT scans over 4 h were performed after intravenous administration of ^18^F-TFB (333–407 MBq) in four female and four male healthy volunteers (35 ± 11 years old). Samples of venous blood and urine were collected over the imaging period and analyzed by ion-chromatography HPLC to determine tracer stability. Vital signs and clinical laboratory safety assays were measured to evaluate safety.

**Results:**

^18^F-TFB administration was well tolerated with no significant findings on vital signs and no clinically meaningful changes in clinical laboratory assays. Left-ventricular blood pool time-activity curves showed a multi-phasic blood clearance of ^18^F-radioactivity with the two rapid clearance phases over the first 20 min, followed by a slower clearance phase. HPLC analysis showed insignificant ^18^F-labeled metabolites in the blood and urine over the length of the study (4 h). High uptakes were seen in the thyroid, stomach, salivary glands, and bladder. Urinary clearance of ^18^F-TFB was prominent. Metabolic stability was evidenced by low accumulation of ^18^F-radioactivity in the bone. Effective doses were 0.036 mSv/MBq in males and 0.064 mSv/MBq in females (*p* = 0.08, not significant).

**Conclusions:**

This initial study in healthy human subjects showed ^18^F-TFB was safe and distributed in the human body similar to other iodide analogs. These data support further translational studies with ^18^F-TFB as NIS gene reporter and imaging biomarker for thyroid cancer and other disease processes that import iodide.

**Electronic supplementary material:**

The online version of this article (10.1186/s13550-017-0337-5) contains supplementary material, which is available to authorized users.

## Background

The sodium/iodide symporter (NIS) is an intrinsic membrane glycoprotein highly expressed in the thyroid gland and other NIS-expressing cells or tissues [1–3]. Human NIS (hNIS) was identified and characterized in 1996 [4, 5], which created various new opportunities using hNIS as a reporter gene in viral therapy investigations and imaging of cell migration and differentiation. Non-invasive monitoring of hNIS expression in normal and hNIS-transfected tissues has been performed by single-photon emission computed tomography (SPECT) with single-photon emitting radioiodides (^123^I and ^125^I) and ^99m^Tc-pertechnetate (^99m^Tc-TcO_4_^−^), whereas positron emission tomography (PET) has used ^124^I-iodide as radiotracer [6]. However, clinical implementation of an ^18^F-fluorine-based radiotracer in positron emission tomography (PET) has lagged, despite of its superior physical decay properties. ^18^F-tetrafluoroborate (^18^F-TFB) has received renewed interest as a promising iodide analog radiotracer for PET imaging in preclinical imaging studies [7–10]. During preparation of this manuscript, the biodistribution and radiation dosimetry estimates of ^18^F-TFB in five thyroid cancer patients were reported [11].

Our laboratory [12] and others [13] have reported high-specific radioactivity syntheses of ^18^F-TFB via the reaction of boron trifluoride (BF_3_) and ^18^F-fluoride. We demonstrated the relationship of ^18^F-TFB-specific radioactivity and PET-delineated radiotracer uptake in NIS-transfected C6-glioma xenograft bearing mice [14], confirming the desirability of high-specific radioactivity to avoid saturation effects at the NIS transporter. In this study, we report PET/CT imaging with ^18^F-TFB in healthy male and female participants to describe radiotracer pharmacokinetics, metabolite analyses, and estimation of radiation dosimetry estimates.

## Methods

### Radiotracer synthesis

^18^F-TFB was prepared and formulated in sterile 0.9% NaCl under Current Good Manufacturing Practice (cGMP) conditions as previously described [12]. Decay-corrected radiochemical yields of 32 ± 2% and radiochemical purities (RCPs) > 98% were obtained. Specific radioactivity of 3.2 ± 1.3 GBq/μmol (at the end of synthesis) was achieved from starting ^18^F-fluoride radioactivity of 20–31 GBq. In vitro radiochemical purity remained > 96% up to 8 h at room temperature.

### Human participants

Approval of the study was obtained from the Mayo Clinic Institutional Review Board, and all participants provided informed consent. All procedures performed in studies involving human participants were in accordance with the ethical standards of the institutional and/or national research committee and with the 1964 Helsinki declaration and its later amendments or comparable ethical standards. Four male (36 ± 14 years) and four female (35 ± 8 years) healthy volunteers were enrolled in the study. Demographic data are shown in Table S1 (Additional file 1). Participants were excluded that had previous diagnosis of cancer or clinically significant cardiovascular, renal, pulmonary, metabolic, and endocrine (thyroid) diseases. Participants were not required to fast prior to the imaging study but instructed to remain well hydrated.

### PET/CT imaging protocol

**Fig. 1:**
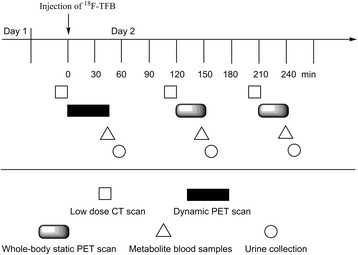
Imaging protocol. Patients were screened on day 1 and imaged by ^18^F-TFB-PET/CT on day 2

The imaging protocol is illustrated in Fig. 1. After voiding of the bladder, participants were positioned in a GE 690XT PET/CT scanner (GE HealthCare, Waukesha, WI) in a supine position with the heart in the center of the axial field of view. Intravenous catheters were placed in both arms for radiotracer injection and blood sampling. Computed tomography scans of the thorax were initially acquired in the preparation for a dynamic PET data acquisition over the heart for the first 45 min following commencement of radiotracer administration. ^18^F-TFB (333–407 MBq) was administered over 1 min into one of the catheters. The frame sequence for initial dynamic PET acquisition was 15 × 4, 8 × 15, 4 × 30, and 8 × 300 s. Following the dynamic heart scan, the participants were allowed a break from the scanner, during which time voided their bladders. Two additional trunk PET/CT scans, from the vertex of the skull to mid-thigh, were performed at 2 and 3.5 h, respectively. The last PET/CT scan was completed ~ 4 h post-injection.

### Measurements in venous blood and urine samples

Urine was collected after each PET/CT scan (approximately 55, 160, 250 min), and venous blood samples were collected at 40, 145, and 235 min, post-injection, as shown in Fig. 1. Blood samples (3–4 mL) were collected in heparinized tubes and placed on ice. The blood samples were centrifuged at 3000 g for 5 min to obtain plasma. Urine was measured for urine volume and radioactivity concentration as measured in a calibrated gamma counter. Analysis for metabolites in plasma and urine was performed by anion chromatography HPLC (Dionex IC-2100, AS19 analytical column 4.7 × 150 mm, eluent 25 mM KOH, sample volume 25 uL, flow rate 1 mL/min, ^18^F-TFB retention time = 7.8 min), allowing estimation of the fraction of radioactivity as non-metabolized (intact) ^18^F-TFB. Radioactivity levels were too low to generate radiochromatograms from the in-line radiation monitor. Based on retention times for ^18^F-fluoride (3 min) and ^18^F-TFB (7.8 min), HPLC effluent was collected at 2–4 min and 6–9 min to represent activity in the form of ^18^F-fluoride and ^18^F-TFB. These fractions were counted in a gamma counter to obtain the percentage of intact ^18^F-TFB.

### Safety measurements for ^18^F-TFB administration

Vital signs (heart rate, systolic and diastolic blood pressures, respiratory rate, and temperature) were measured prior to ^18^F-TFB administration and at 45 and 240 min post-injection. Venous blood samples were taken prior to ^18^F-TFB administration and at ~ 240 min post-injection for a panel of clinical laboratory tests to evaluate the safety of the radiotracer administration.

### PET/CT image analysis

All PET scans images were reconstructed using 3-dimensional ordered subset expectation maximization (3D OSEM) and time-of-flight reconstruction. Volumes of interest (VOIs) of organs were manually drawn based on the CT imaging data using PMOD software (Ver. 3.5). The VOIs included the whole organ. Standardized Uptake Values (SUVs) normalized to body weight were then calculated for each VOI. Time-activity curves (TACs) were evaluated from the initial dynamic scan over the heart for left-ventricular blood pool, lungs, and liver regions.

### Pharmacokinetic analysis

Pharmacokinetic analysis was performed by fitting the left-ventricular blood pool time-activity curves (in units of SUV) to a 3-exponential model: *x* = *A*_1_ *e* ^−*k*^_1_^*t*^ + *A*_2_ *e* ^−*k*^_2_^*t*^ + *A*_3_ *e* ^−*k*^_3_^*t*^ using least squares regression. The time-activity curves were shifted in time to set the peak value at time *t* = 0.

### Radiation dosimetry estimation

Radiation dosimetry estimates were calculated from organ residence times using OLINDA software (Ver. 1.1), assuming a bladder voiding interval of 3.5 h. To compute organ residence times, the decay correction was removed from the VOI data. Since not all tissues were represented in the initial dynamic PET scan over the heart, the unrepresented tissues were assumed to have static distribution of ^18^F-TFB from time of injection to the measurement at 2 h. Thus, radioactivity concentrations in each organ were obtained from the 2 h time point data corrected for radioactive decay by the multiplicative factor *e* ^*λ*(120 − *t*)^, where *t* is time in minutes post-injection and *λ* (0.00632 min^−1^) is the decay constant for ^18^F. Gender-specific organ masses for the conversion of SUV to disintegrations per organ per unit radioactivity administered (Bq-hr./Bq) were taken from Schlein et al. [15].

### Statistics

Descriptive statistics are provided as mean ± standard deviation (SD) or counts and percentages. Comparisons between groups by gender or between subjects at two different scans used Wilcoxon rank sum and signed rank test, respectively. Comparisons for vital statistics used the Friedman rank sum test. Statistical significance was defined as *p* values < 0.05. Results have not been adjusted for multiple comparisons. Analyses were performed in R (version 3.2.3; Vienna, Austria).

## Results

The cohort was composed of four female and four male subjects, 35 ± 11 years of age, with BMI of 28.3 ± 6.9 kg/m^2^. The mean ± SD injected doses were 333–407 MBq. With the exception of height (*p* = 0.02), no baseline differences were observed between genders in age, weight, BMI, diabetic status (none were diabetic), or injected dose.

### Pharmacokinetics of ^18^F-TFB

**Fig. 2:**
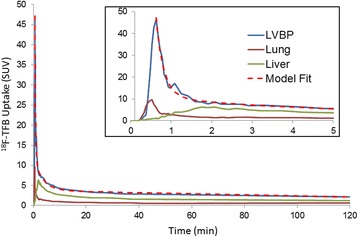
Time-activity curves of ^18^F-TFB in healthy male participant. *LVBP* left-ventricular blood pool. Inset shows first 5 min of data. The LVBP time-activity curves were fit by a 3-exponential model

The initial 45-min dynamic PET/CT scan over the heart allowed evaluation of the early kinetics of ^18^F-TFB in the left-ventricular blood pool (LVBP) as in indication of the pharmacokinetics of this radiotracer (Table 1). Figure 2 shows the time-activity curves in LVBP, lung, and liver in a representative male participant. Peak values of radioactivity concentration were seen at ~ 1 min for LVBP and lung, whereas the peak in liver was at ~ 2 min post-administration of ^18^F-TFB. The blood clearance kinetics following the peak activity were fit to a 3-exponential model as shown in Fig. 2. Parameter estimates are shown in Table 2. Blood clearance were fit well by a 3-exponential model with a rapid clearance phase (*T*_½_ ~ 5 s), a moderately rapid clearance phase (*T*_½_ ~ 1.5 min), and a slower clearance phase (*T*_½_ > 30 min) proceeding to the end of the 4-h measurement period. There were no significant gender differences in the pharmacokinetic parameters. The slow clearance component (*A*_3_) represented approximately 14 ± 5% and 20 ± 9% of peak activity in males and females, respectively, with no significant gender difference. Continued radiotracer washout was also seen in the lung and liver.

**Table 1:** Pharmacokinetics of ^18^F-TFB in healthy participants

Parameter^a^	3-exponential fit parameter estimates	
	Males	Females
*A*_1_ (SUV)	27.9 ± 9.5	23.2 ± 29.3
*k*_1_ (min^−1^)	7.6 ± 2.8	9.7 ± 6.8
*A*_2_ (SUV)	8.7 ± 3.0	6.8 ± 2.0
*k*_2_ (min^−1^)	0.64 ± 0.36	0.44 ± 0.09
*A*_3_ (SUV)	5.9 ± 1.6	5.1 ± 0.3
*k*_3_ (min^−1^)	0.023 ± 0.023	0.016 ± 0.005

Values are mean ± SD (*n* = 4)

^a^SUV values derived from left-ventricular blood pool were fit to 3-exponential model: $x={A}_1{e}^{-{k}_1t}+{A}_2{e}^{-{k}_2t}+{A}_3{e}^{-{k}_3t}$

**Table 2:** Metabolite analysis of ^18^F-TFB in plasma and urine of healthy participants

		40 min^a^		145 min^a^		235 min^a^		Accumulative	
		Males	Females	Males	Females	Males	Females	Males	Females
Whole blood (SUV)		3.0 ± 0.2	3.1 ± 0.9	2.1 ± 0.2	1.7 ± 0.4	1.7 ± 0.2	1.4 ± 0.4	–	–
Plasma (SUV)		3.8 ± 0.2	3.8 ± 1.0	2.8 ± 0.3	2.1 ± 0.5	2.0 ± 0.1	1.7 ± 0.5	–	–
Urine (%dose)		15 ± 2^b^	16 ± 3	13 ± 3	18 ± 3	10 ± 2^b^	12 ± 2	40 ± 5^b^	46 ± 7
%Intact ^18^F-TFB	Plasma	97 ± 2	97 ± 3^c^	−^d^	−^d^	−^d^	−^d^	–	–
	Urine	96 ± 2	98 ± 1	97 ± 2	98 ± 1	98 ± 1	97 ± 2	–	–

Values are mean ± SD (*n* = 4)

^a^Time points are shown for blood samples; urine samples were collected ~ 10 min later

^b^No urine in third collection of one male subject

^c^One plasma sample was not used because precipitate was observed in the HPLC analysis

^d^Metabolite data were not obtained in second and third plasma samples analysis because the low radioactivity levels were below detection limit

### Metabolite analysis

HPLC metabolite analysis of plasma samples at 40 min p.i. showed > 97% of activity to be in the form of metabolically intact ^18^F-TFB (Table 2). Similarly, all urine samples taken from urine collections at approximately 50, 160, and 250 min showed > 97% of activity in the form of ^18^F-TFB. The accumulated percentage of administered ^18^F-TFB in the urine at the end of study (~ 250 min) was 40 ± 5% for males and 46 ± 7% for females. Thus, renal clearance of non-metabolized ^18^F-TFB is a major excretion pathway for ^18^F-TFB. There was no difference between genders in the percent dose excreted in urine at each of the three measurement times or in total percent excreted (*p* = 0.56, 0.25, 0.48, 0.29). Overall, both whole blood and plasma SUV values significantly decreased over time, both from 45 to 145 min post-injection and again between 145 and 235 min (*p* = 0.008, all pairwise comparisons).

### Biodistribution of ^18^F-TFB

**Fig. 3:**
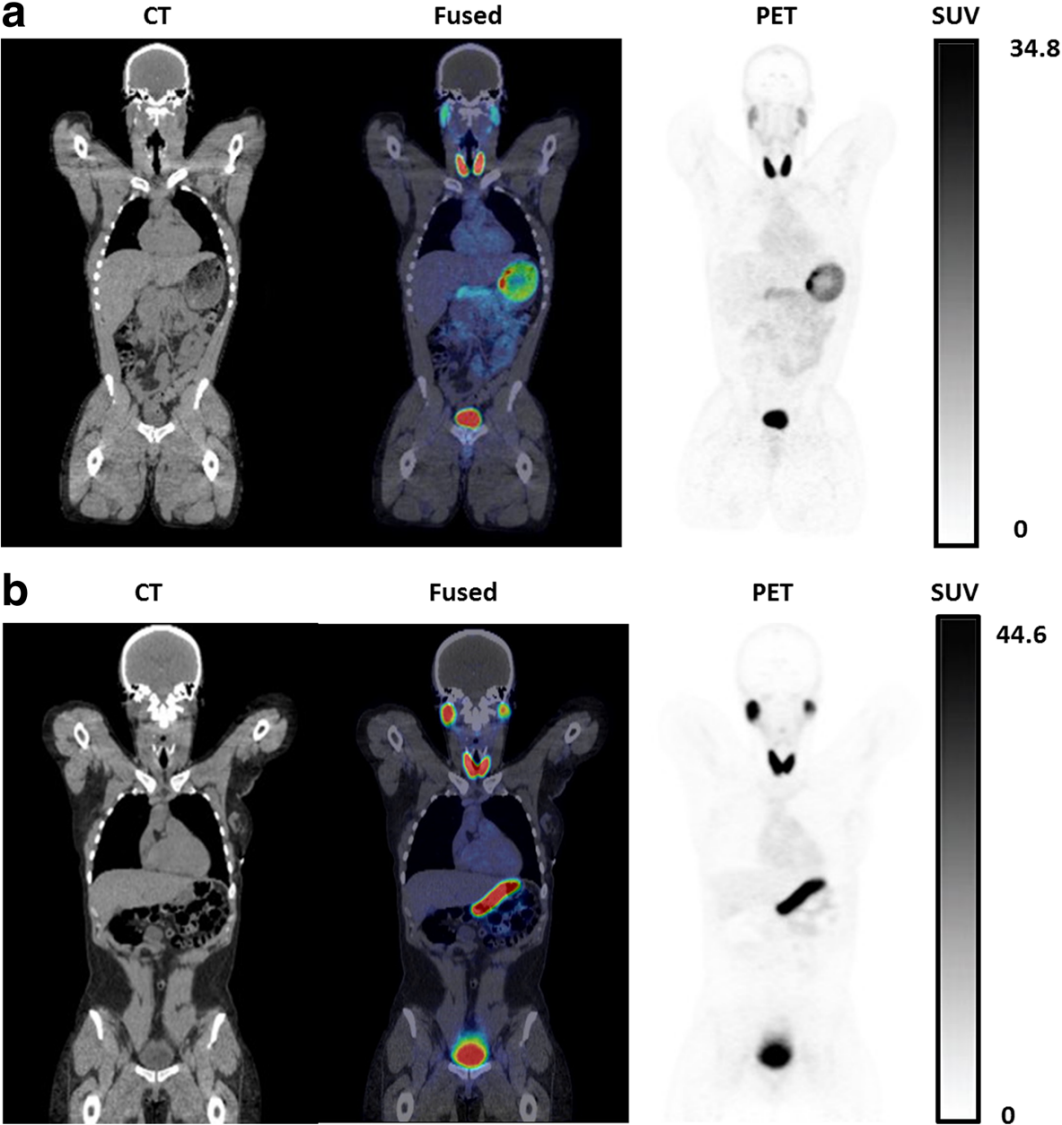
Coronal PET/CT images of ^18^F-TFB in healthy male (**a**) and female (**b**) participants at 2 h post-injection. Physiologic distribution of ^18^F-TFB is seen in the thyroid, salivary glands, stomach, and intestines. Prominent excretion of radioactivity is seen in the urinary bladder

Representative images from the whole-body PET/CT scan at 2 h post-injection are shown in Fig. 3, and the biodistribution data are summarized in Table 3. Robust uptake of ^18^F-TFB was observed in the thyroid, stomach, salivary glands, and kidneys. Prominent clearance of tracer through the kidneys to bladder was also observed. Minor differences were observed in the SUV values between the first (2 h) and second (3.5 h, Figure S1, Additional file 1) whole-body PET/CT scans, showing the tracer distribution to be stable after 2 h. All organs showed small but positive changes in SUVs between the two whole-body PET/CT scans; these changes were significant except in the breasts, intestines, pancreas, and stomach. In females, a low but moderate uptake was observed in breast tissue. Relative increases in bone uptake from 2 to 3.5 h ranged widely from 2 to 60% in individual participants, possibly due to individual differences in defluorination. However, since bone uptake was > 10-fold lower than for the tissues with high expression of NIS (thyroid, stomach, salivary glands), bone uptake was not remarkable in the PET/CT images.

**Table 3:** PET/CT-derived distribution of ^18^F-TFB in healthy participants

Organ	2 h post-injection (SUV)		3.5 h post-injection (SUV)	
	Males	Females	Males	Females
Bone	0.80 ± 0.33	0.74 ± 0.20	1.4 ± 0.5*	1.3 ± 0.9*
Brain	0.39 ± 0.14	0.42 ± 0.15	0.74 ± 0.26*	0.61 ± 0.34*
Breast	–	2.8 ± 0.4	–	3.9 ± 1.4
Gallbladder	2.7 ± 1.5	3.7 ± 1.0	4.2 ± 1.7*	5.0 ± 1.6*
Intestines	2.9 ± 1.0	5.0 ± 2.7	4.4 ± 1.0	6.0 ± 3.1
Kidney	7.0 ± 2.5	5.6 ± 0.7	11 ± 3*	6.3 ± 2.4*
Liver	2.3 ± 1.0	2.7 ± 0.5	3.6 ± 1.2*	3.5 ± 1.6*
Lung	1.0 ± 0.4	1.2 ± 0.4	1.7 ± 0.6*	1.5 ± 0.8*
Muscle	0.71 ± 0.31	0.65 ± 0.35	0.96 ± 0.31*	0.78 ± 0.54*
Myocardium	3.2 ± 0.9	2.8 ± 0.5	5.0 ± 0.9*	3.8 ± 1.8*
Pancreas	3.4 ± 0.7	3.1 ± 1.7	6.2 ± 2.1	3.0 ± 1.2
Parotid	11 ± 9	20 ± 11	16 ± 13*	25 ± 15*
Spleen	4.1 ± 1.2	4.3 ± 1.0	6.3 ± 1.1*	5.5 ± 2.8*
Stomach	33 ± 15	72 ± 10	70 ± 32	51 ± 18
Thyroid	55 ± 31	50 ± 11	82 ± 42*	58 ± 12*

Values are mean ± SD (*n* = 4)

*p < 0.05 versus 2 h

A significant difference was observed between genders in TFB uptake at scan 1 for the stomach, with females showing higher SUVs (females 72.2 ± 10.2, males 33.4 ± 14.7, *p* = 0.021). Between scan 1 and scan 2, there was also a significant difference between genders in the change in SUV values for the stomach (females  21 ± 17.4, males 36.4 ± 18.9, *p* = 0.021), as well as the pancreas (females 0.049 ± 1.14, males 2.74 ± 1.40, *p* = 0.043). At scan 2, there were no significant differences observed between genders. In the cohort as a whole, all organs showed a positive change in SUVs between scan 1 and scan 2; these changes were significant except in the breast, intestines, pancreas, and stomach.

### Radiation dosimetry estimates

Table 4 shows organ residence times derived from the biodistribution data. Bladder residence time was estimated by the bladder emptying model within the OLINDA software, assuming a 3.5-h bladder voiding period. Estimated organ absorbed doses are shown in Table 5. The dose-critical organ is the thyroid, with dose estimates of 0.26 and 0.36 mSv/MBq in males and females, respectively. The prominent excretion through the bladder resulted in moderately high doses to the bladder wall, with doses depending on voiding frequency. Effective doses are shown in Table 6. Effective dose was higher in females (0.064 mSv/MBq) relative to males (0.036 mSv/MBq), but the difference did not reach statistical significance (*p* = 0.08). No significant differences in organ residence times were observed between genders for any organ. Significant gender differences in organ effective doses were observed for the gallbladder wall, lower large intestine wall, small intestine wall, stomach wall, lungs, red marrow cells, spleen, and urinary bladder wall (Table 5).

**Table 4:** Organ residence times of ^18^F-TFB in healthy participants

Organ	Residence time males (min)	Residence time females (min)
Bone	4.6 ± 1.3	3.1 ± 0.3
Brain	1.2 ± 0.4	1.3 ± 0.2
Breast	–	2.8 ± 0.9
Gallbladder	0.06 ± 0.03	0.09 ± 0.04
Intestine	5.7 ± 1.9	13 ± 7
Kidney	4.2 ± 1.2	3.9 ± 0.8
Liver	8.3 ± 3.9	10 ± 2
Lung	2.1 ± 0.7	2.4 ± 0.2
Muscle	36 ± 14	26 ± 8
Myocardium	2.1 ± 0.6	1.8 ± 0.3
Pancreas	0.7 ± 0.3	0.58 ± 0.08
Parotid	1.7 ± 1.1	3.3 ± 1.2
Spleen	1.5 ± 0.4	1.6 ± 0.2
Stomach	11 ± 5	24 ± 12
Thyroid	2.0 ± 0.7	2.3 ± 0.9
Bladder	17 ± 2	19 ± 4

Values are mean ± SD (*n* = 4)

No significant (*p* < 0.05) differences were observed in organ residence times between genders

**Table 5:** Estimated absorbed radiation dose for ^18^F-TFB (mSv/MBq)

Organ	Dose (mSv/MBq)	
	Males	Females
Adrenals	0.008	0.012
Brain	0.004	0.005
Breast	0.002	0.028
Gallbladder wall*	0.012	0.022
Lower large intestine wall*	0.009	0.015
Small intestine wall*	0.027	0.066
Stomach wall*	0.076	0.184
Upper large intestine wall	0.045	0.109
Heart wall	0.024	0.030
Kidney	0.049	0.052
Liver	0.021	0.033
Lung*	0.009	0.014
Muscle	0.008	0.011
Ovaries	–	0.020
Pancreas	0.034	0.043
Red marrow*	0.005	0.008
Osteogenic cells	0.008	0.009
Skin	0.003	0.004
Spleen*	0.033	0.047
Testes	0.005	–
Thymus	0.004	0.005
Thyroid	0.26	0.36
Urinary bladder wall	0.14	0.22
Uterus	–	0.022
Total body	0.008	0.011

*p < 0.05 between genders

**Table 6:** Effective dose for ^18^F-TFB in healthy participants

	Males	Females
Effective dose (mSv/MBq)	0.036	0.064

### ^18^F-TFB safety data

Vital signs (heart rate, diastolic and systolic blood pressures, and respiratory rate) were monitored before ^18^F-TFB administration and throughout the PET/CT imaging period. No significant changes in the vital signs were found after ^18^F-TFB administration (Additional file 1: Table S2). No significant differences between pre- and post-injection measurements were observed in any vital signs (heart rate (*p* = 0.14), blood pressure (DBP, *p* = 0.41; SBP, *p* = 0.88), respiratory rate (*p* = 0.47), and temperature (*p* = 0.43)). A panel of clinical laboratory blood tests was measured to assess for effects of ^18^F-TFB administration on blood chemistries, including electrolytes, and liver and kidney functional tests. While some changes in lab values between pre- and post-injections were statistically significant, the magnitudes of these changes were small, and no clinically meaningful differences were observed (Additional file 1: Table S3).

## Discussion

The aim of this study was to evaluate the safety, pharmacokinetics, metabolism, biodistribution, and radiation dosimetry of high-specific radioactivity ^18^F-TFB in eight healthy human participants. The tracer was well tolerated, and no adverse effects were noted. hNIS is known to be highly expressed in certain tissues (thyroid, breasts, stomach, and salivary glands) as well as hNIS-transfected tissues. Thus, non-invasive PET imaging of hNIS activity could be used to facilitate the treatment of thyroid and breast cancer and gene therapies that employ hNIS as a reporter gene. In principle, the ^18^F-TFB PET method may also enable quantitative estimation of hNIS activity in tissues. Therefore, it may be useful to monitor changes over time for understanding the progression of disease and serial assessments of therapy response.

Since only high-specific radioactivity ^18^F-TFB was administered in this study, we did not explore the effects of specific radioactivity over a broader range. However, in our previous preclinical study with NIS-transfected C6 glioma xenografts in mice, we showed that as specific radioactivity was decreased such that TFB administration levels exceeded ~ 0.5 mg/kg, there was decreases seen in both thyroid and tumor uptake. Since thyroid uptake levels in the healthy participants in this study were very high, it is inferred that the specific radioactivity levels were sufficient to avoid any saturation effects do to administered TFB mass.

The regional distribution of ^18^F-TFB in healthy participants was found to be consistent with known hNIS expression levels throughout the body tissues. The slow accumulation of ^18^F-radioactivity seen in SUV values between the 2 and 3.5 h imaging time points could be evidence of a minor degree of radiotracer defluorination, but since routine ^18^F-TFB PET images will likely be acquired in the 1-2 h post-injection period, the impact of this accumulation is of minor significance. Indeed, bone uptake was not qualitatively remarkable in either the 2 or 3.5 h images. Overall, the biodistribution data confirm ^18^F-TFB to be an excellent iodide analog radiotracer with an excellent in vivo stability. The blood clearance kinetics were found to be adequately fit by a 3-exponential model, with the slowest component representing 14–20% of peak activity and showing a half-clearance time of about 30 min. No gender differences were noted in the pharmacokinetics. For purposes of static PET/CT imaging, an uptake period of 45–60 min should allow good clearance of tracer from blood and tissues with low expression of NIS.

NIS expression in breast cancer has been noted in several reports [16, 17]. The normal uptake of ^18^F-TFB in breasts of healthy female participants was found to be above adjacent background, showing SUVs in the 2.8–3.9 range (Table 2). Further studies will be required to determine whether ^18^F-TFB uptake in primary or metastatic breast tumors will be sufficiently higher relative to healthy breast tissue to allow evaluation of breast cancer.

Gene therapy has been extensively applied in various liver diseases, such as liver cancer and hepatitis [18, 19]. In a previous ^18^F-TFB PET/CT imaging of Pig Y842 [20], in which hepatocytes were transduced with lentiviral vectors expressing the therapeutic fumarylacetoacetate hydrolase (FAH) and the reporter NIS genes, significant ^18^F-TFB uptake was demonstrated in the liver, thus allowing non-invasive evaluation of repopulating cluster of FAH^+^, while no liver uptake in the control pigs. The result shows ^18^F-TFB may serve as a promising tracer for human liver gene therapy. The low physiologic uptake of ^18^F-TFB in the liver is advantageous for monitoring of viral therapies with NIS reporter genes.

The estimated radiation doses were highest in the thyroid, urinary bladder wall, lower large intestine wall, small intestine wall, upper large intestine wall, heart wall, kidneys, liver, pancreas, and spleen, but on par with other ^18^F-labeled radiopharmaceuticals and appropriate for clinical use. Further decrease in bladder wall doses can be realized with good hydration and more frequent voiding of the bladder. Estimated effective doses were 0.036 mSv/MBq in males and 0.064 mSv/MBq in females, showing a trend of higher effective dose in females that was not statistically significant (*p* = 0.08). The effective dose estimates for ^18^F-TFB were about twofold higher than those reported for ^18^F-FDG [21]. Our data on the biodistribution and dosimetry estimates for ^18^F-TFB are in general agreement with the results very recently published by O’Doherty et al. [11] in five patients with thyroid cancer. In that study, 2 male and 3 female subjects were studied, and the results from both genders were pooled. The specific radioactivity of their ^18^F-TFB preparations (24 ± 13 MBq/μg) was similar to the specific radioactivity obtained in this study. Quinn et al. [21] investigated ^18^F-FDG dosimetry estimates in 95 men and 88 women, also finding higher but not statistically different effective doses in the female group. For comparison with other NIS-imaging radiotracers, the effective doses (mSv/MBq) of radioiodines ^123^I (0.23–0.31), ^124^I (13–18), and ^131^I (22–29) are significantly higher than the estimates for ^18^F-TFB (0.051 mSv/MBq for averaged male and females), whereas that of ^99m^Tc-TcO_4_^−^ is lower (0.013 mSv/MBq) [22]. The higher effective doses of the radioiodines can be attributed to differences in biodistribution secondary to (1) different affinity of iodine for NIS transporters; (2) organification of iodine within thyroid, incorporation into thyroid hormone molecules, and subsequent redistribution of these metabolites; and/or (3) higher urinary excretion of ^18^F-TFB and ^99m^Tc-TcO4^−^.

This study had the limitation that the choice to acquire early dynamic imaging over the heart to measure radiotracer pharmacokinetics with high temporal resolution from left-ventricular blood pool regions precluded acquisition of whole-body biodistribution information in the first hour after injection. To allow for collection of urine and give the subjects a rest break from the scanner, the first of two trunk PET/CT scans were commenced at 2 h post-injection. For calculation of radiation dosimetry estimates, the distribution of radioactivity in the regions not represented in the initial dynamic data was assumed to be static (accounting for radioactive decay) between the time of injection and the 2-h time point. This assumption may lead to error in the dosimetry estimates, although the very slow blood clearance kinetics after 1 h (Fig. 2) and the very similar biodistribution pattern seen and 2 and 3.5 h PET/CT scans (Table 2) suggests that there is a minimal ^18^F-TFB redistribution in the body once the tracer is taken up in tissues. Indeed, O′ Doherty et al. [11] recently acquired serial whole-body images of ^18^F-TFB immediately after IV injection in five thyroid cancer patients, showing there was very similar biodistribution of radiotracer from about 15 min to 2 h with major uptake organs being salivary glands, thyroid, stomach, and bladder. Their dosimetry estimates were very similar to those obtained in the present study.

## Conclusions

^18^F-TFB was found to be well tolerated by the participants in the study. The rapid pharmacokinetics, absence of metabolism, and specific biodistribution to NIS-expressing tissues of high-specific radioactivity ^18^F-TFB in healthy human participants support its use as an iodide analog radiotracer for evaluation of thyroid and breast cancers and monitoring of gene therapies that employ the hNIS reporter gene. The radiation dosimetry estimates are on par with other ^18^F-labeled radiopharmaceuticals with prominent renal excretion (e.g., ^18^F-FDG) and are acceptable for clinical imaging purposes.

## Additional file

Additional file 1: Supplemental material. (DOCX 197 kb)
