# Supplementary material for: Safety, pharmacokinetics, metabolism and radiation dosimetry of 18F-tetrafluoroborate (18F-TFB) in healthy human subjects
Source: EJNMMI Res. 2017 Oct 27;7:90. doi: 10.1186/s13550-017-0337-5 (PMC5660009; doi:10.1186/s13550-017-0337-5)
Supplement: Additional file 1: — Supplemental material. (DOCX 197 kb) [file 13550_2017_337_MOESM1_ESM.docx]

**Supplemental Material**

**Safety, Pharmacokinetics, Metabolism and Radiation Dosimetry of ^18^F-Tetrafluoroborate (^18^F-TFB) in Healthy Human Subjects**

Huailei Jiang^1^, Nicholas R. Schmit^1^, Alex R. Koenen^1^, Aditya Bansal^1^, Mukesh K. Pandey^1^, Robert B. Glynn^1^, Bradley J. Kemp^1^, Kera L. Delaney^1^, Angela Dispenzieri^2^, Jamie N. Bakkum-Gamez^3^, Kah-Whye Peng^4^, Stephen J. Russell^4^, Tina M. Gunderson^5^ Val J. Lowe^1^, Timothy R. DeGrado^1^

^1^Department of Radiology, Mayo Clinic, Rochester MN USA

^2^Department of Medicine, Mayo Clinic, Rochester MN USA

^3^Department of Obstetrics and Gynecology, Mayo Clinic, Rochester MN USA

^4^Department of Molecular Medicine, Mayo Clinic, Rochester MN USA

^5^Department of Clinical Statistics, Mayo Clinic, Rochester MN USA

Corresponding author:

Dr. Timothy R. DeGrado

Department of Radiology

Mayo Clinic

200 First St. SW

Rochester, MN 55905

E-mail: [degrado.timothy@mayo.edu](mailto:degrado.timothy@mayo.edu)

**Table S1**. Demographic data on participants

|  | Females | Males | p value |
| --- | --- | --- | --- |
| N Age (y) Height (cm) Wt. (kg) BMI Injected dose (MBq) | 4  35 ± 8  160 ± 6  71 ± 32  27 ± 10  377 ± 17 | 4  36 ± 14  183 ± 5  99 ± 16  30 ± 3  389 ± 10 | 0.77 0.021 0.149 0.248 0.248 |

**Table S2.** Vitals data pre- and post-injection (40 min, 240 min). Values are presented as mean ± standard deviation (n = 8). Friedman's test was performed.

| Vital | Pre-injection | 40 min | 240 min | p-value |
| --- | --- | --- | --- | --- |
| DBP | 85 ± 9 | 84 ± 9 | 86 ± 10 | 0.41 |
| HR | 92 ± 18 | 90 ± 19 | 96 ± 26 | 0.14 |
| RR | 18 ± 5 | 16 ± 3 | 16 ± 3 | 0.47 |
| SBP | 128 ± 15 | 128 ± 16 | 132 ± 18 | 0.88 |
| Temp | 36.5 ± 0.3 | 36.4 ± 0.5 | 36.6 ± 0.3 | 0.43 |

**Table S3**. Laboratory values pre- and post-injection. Values are presented as mean± standard deviation (n = 8). Wilcoxon signed rank tests were performed for each lab.

| Lab name | Pre-Injection Mean±SD | Post-Injection Mean±SD | Change in lab value | p-value |
| --- | --- | --- | --- | --- |
| Albumin | 4.45 ± 0.382 | 4.55 ± 0.283 | 0.1 ± 0.193 | 0.17 |
| Alk Phosphatase | 62.9 ± 16.3 | 65.2 ± 14.5 | 2.38 ± 2.92 | 0.08 |
| ALT (GPT)(S) | 26.4 ± 16.2 | 28 ± 15 | 1.56 ± 3.46 | 0.30 |
| Basophils_AD | 0.047 ± 0.038 | 0.179 ± 0.363 | -0.01 ± 0.011 | 0.12 |
| Bicarbonate, P/S | 25.1 ± 2.53 | 25.2 ± 2.05 | 0.125 ± 2.95 | 0.84 |
| Bilirubin, Total, S | 0.5 ± 0.262 | 0.5 ± 0.288 | 0 ± 0.0756 | 1.00 |
| BUN | 13 ± 3.25 | 12.4 ± 2.92 | -0.625 ± 0.744 | 0.12 |
| Calcium | 9.29 ± 0.439 | 9.53 ± 0.492 | 0.238 ± 0.13 | 0.01 |
| Chloride | 102 ± 1.93 | 100 ± 2.07 | -1 ± 1.2 | 0.09 |
| Cholesterol(S) | 177 ± 41.4 | 185 ± 41.7 | 7.25 ± 7.46 | 0.02 |
| Creatinine (w/eGFR) | 0.8 ± 0.131 | 0.775 ± 0.167 | -0.025 ± 0.07 | 0.50 |
| Eosinophils_AD | 0.152 ± 0.095 | 0.167 ± 0.123 | 0.004 ± 0.027 | 0.91 |
| Erythrocytes | 4.58 ± 0.47 | 4.67 ± 0.471 | 0.064 ± 0.08 | 0.09 |
| Serum Glucose | 91.4 ± 9.3 | 92.4 ± 5.24 | 1 ± 5.88 | 1.00 |
| Hematocrit | 40.6 ± 2.96 | 41.3 ± 2.97 | 0.514 ± 0.696 | 0.16 |
| Hemoglobin | 13.8 ± 1.39 | 14.3 ± 1.33 | 0.329 ± 0.214 | 0.03 |
| IgA(S) | 209 ± 89.6 | 218 ± 91 | 9.25 ± 9.71 | 0.08 |
| IgG(S) | 1081 ± 128 | 1095+03 ± 128 | 14.2 ± 46.3 | 0.47 |
| IgM(S) | 106 ± 48.6 | 111 ± 55.1 | 5.25 ± 9.98 | 0.22 |
| Leukocytes | 13.6 ± 22 | 6.37 ± 1.08 | -8.19 ± 23 | 0.98 |
| Lymphocytes_AD | 2.04 ± 0.605 | 2.12 ± 0.622 | 0.087 ± 0.208 | 0.41 |
| Magnesium(S) | 2.01 ± 0.084 | 2.09 ± 0.0641 | 0.075 ± 0.046 | 0.03 |
| MCH | 30.2 ± 1.26 | 30.6 ± 1.42 | 0.343 ± 0.346 | 0.05 |
| MCHC | 34 ± 1.15 | 34.5 ± 0.995 | 0.4 ± 0.44 | 0.06 |
| MCV | 88.9 ± 3.55 | 88.6 ± 3.72 | -0.129 ± 0.541 | 0.28 |
| Monocytes | 0.616 ± 0.514 | 0.659 ± 0.675 | 0.0157 ± 0.156 | 0.81 |
| Neutrophils | 3.24 ± 0.861 | 3.63 ± 1.02 | 0.497 ± 0.825 | 0.16 |
| Phosphorus | 3.19 ± 0.439 | 3.38 ± 0.365 | 0.188 ± 0.259 | 0.09 |
| Platelet Count | 262 ± 38.7 | 243 ± 55.6 | -14.7 ± 19.7 | 0.03 |
| Potassium, S | 4.39 ± 0.253 | 4.33 ± 0.18 | -0.043 ± 0.199 | 0.75 |
| Protein,Total | 6.92 ± 0.575 | 7.16 ± 0.358 | 0.237 ± 0.346 | 0.12 |
| RBC Distrib Width | 13 ± 0.572 | 12.9 ± 0.615 | -0.114 ± 0.146 | 0.16 |
| Sodium, S | 139 ± 1.77 | 139 ± 2.17 | 0.125 ± 2.03 | 1.00 |
| Triglycerides(S) | 174 ± 105 | 186 ± 113 | 12.2 ± 29.1 | 0.30 |
| Tryptase | 6.6 ± 5.77 | 6.36 ± 5.48 | -0.237 ± 0.431 | 0.34 |
| Uric Acid(S) | 5.38 ± 2.28 | 5.33 ± 2.33 | -0.05 ± 0.169 | 0.56 |


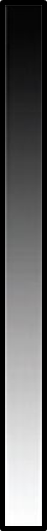


35.5

0


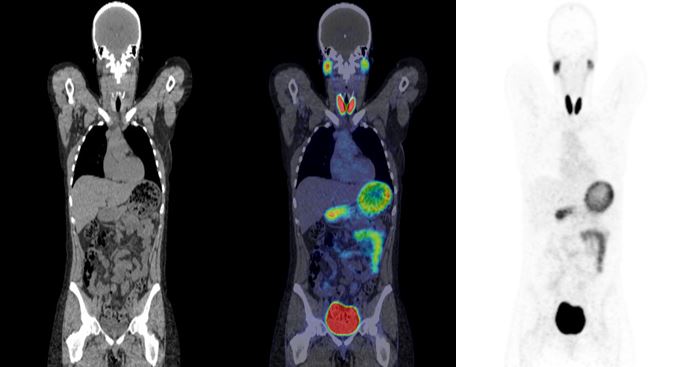

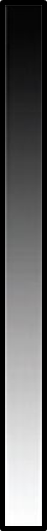


36.6

0

CT

Fused

PET


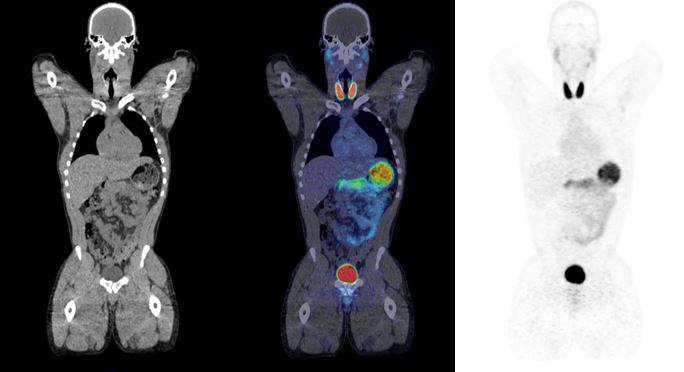


**CT**

**Fused**

**PET**

**SUV**

**A**

**CT**

**Fused**

**PET**

**SUV**

**B**

**Figure S1**. Coronal PET/CT images of ^18^F-TFB in healthy male (A) and female (B) participants at 3.5 h post-injection. Minor bone uptake was found. Food was taken before the 3.5 h imaging for the female subject.
